# A long-term air quality and meteorological coupling dynamics in Abeokuta, Nigeria

**DOI:** 10.1371/journal.pgph.0006683

**Published:** 2026-08-07

**Authors:** Nathaniel Oluwatimileyin Adeniji, Jacob Adebayo Akinpelu, Francis Olatunbosun Aweda, Solomon Oluwadara Adeola, Olusanya Odunayo Jegede

**Affiliations:** Physics Programme, Bowen University, Iwo, Osun State, Nigeria; US Environmental Protection Agency, UNITED STATES OF AMERICA

## Abstract

This study examines the dynamics of ambient air pollutants and their meteorological determinants in Abeokuta, Nigeria. This study averaged 12 years of hourly data (2012–2023) for PM_2_._5_, PM₁₀, NO_2_, SO_2_, CO, and O_3_, alongside six meteorological variables: temperature, relative humidity, pressure, wind speed, wind direction, and rainfall. Specifically, Particulate matter (PM_2.5_ and PM_10_) were the most dominant pollutants, consistently surpassing World Health Organisation (WHO) standards and reaching their highest concentrations during the Harmattan dry season, typically occurring between December and March. Regression analysis demonstrated moderate to strong positive correlations with temperature (R² = 0.48–0.52), while also indicating negative relationships with rainfall and humidity, thereby validating the impacts of thermal stagnation and wet scavenging processes. Furthermore, fluctuations in atmospheric pressure contributed to the buildup of pollutants during periods of stagnant air. Gaseous pollutants showed relatively weaker sensitivity; however, levels of NO_2_ and CO increased during the hot, arid months, while O_3_ levels rose in the late dry season. The Aggregate Air Quality Index (AAQI) values fluctuated between 39 and 51, thereby designating the air quality as “moderate.” Furthermore, the Health-Risk Adjusted AQI (HAQI) revealed heightened relative risks, especially concerning PM_2_._5_ exposure, with seasonal maxima exceeding 90. These findings highlight the cumulative health impacts of particulate matter, even when gaseous pollutants remain within permissible thresholds. The results highlight the necessity for seasonally adjusted mitigation measures, including dust suppression, emission controls, and cleaner combustion technologies. Furthermore, they advocate for the incorporation of Health-Adjusted Quality Index (HAQI) metrics within air quality management systems.

## Introduction

Air pollution is a major environmental health risk and is associated with increased morbidity and mortality from cardiovascular and respiratory diseases worldwide [1]. In places where cities are growing quickly, traffic emissions, biomass burning, industry, power generation, and dust that moves around during the year can all cause exposure. These effects are greatly influenced by seasonal wet-dry patterns and can cause reduced air quality, impairs visibility and hazardous to health all at once [1]. These interactions complicate the development of effective air-quality management and climate-adaptation strategies in the region. Studies in have demonstrated that temperature, boundary layer dynamics, rainfall scavenging and precursor chemistry substantially affect the variability of particulate matter and ozone [2,3,4]. Similar evidence from southern Africa highlights the existence of mixed urban -industrial particulate sources and the importance of health-risk composite indices [5,6].

Studies conducted previously in Nigeria and other tropical urban centres have consistently reported significant seasonal variations in air pollutant levels due to meteorological and emission conditions. In Nigeria, high concentrations of particulate matter in the dry season have been attributed to transport of Harmattan dust, biomass burning, traffic emissions and the resuspension of road dust, while wet season rainfall enhances pollutant removal via atmospheric scavenging and increased dispersion [7,8,9]. Similar seasonal behaviour has been reported across African and Asian cities where temperature, atmospheric stability, boundary-layer dynamics and precipitation strongly influence pollutant accumulation and dilution [10,11,12]. However, many previous investigations have focused on individual pollutants, short-term observations or limited evaluations of meteorological controls. Long-term studies comprehensively integrating multiple pollutants, meteorological drivers, health-risk assessment indices and predictive modelling are still scarce, especially for fast urbanising West African cities where the local anthropogenic emissions as well as the regional intrusions of the Harmattan dust can play a role. To this end, this study presents an updated twelve-year assessment (2012–2023) of pollutant-meteorology interactions in Abeokuta including AQI, AAQI, HAQI and concentration projections to facilitate evidence-based air-quality management and public-health planning.

Harmattan which is a dry and dusty north-easterly trade wind that originates from the Sahara Desert and typically occurs between November and March [13], is characterised by low humidity, reduced visibility, elevated aerosol loading, and intense dust transport, which significantly increase ambient concentrations of particulate matter such as PM_2_._5_ and PM₁₀ across the region  [14]. During Harmattan episodes, suspended mineral dust particles can travel thousands of kilometres across West Africa, thereby intensifying respiratory exposure risks and contributing substantially to seasonal deterioration in air quality  [15].

The movement of Harmattan dust in West Africa mixes with emissions from cities, which raises the risk of exposure [16], observations from Nigeria confirm both regional and local sources of particulate pollution [17]. This study analysed the monthly concentrations of PM_2.5_, PM_10_, SO_2_, NO_2_, CO and O_3_ in Abeokuta from 2012 to 2023, alongside six meteorological variables.

In the present study, Air Quality Index (AQI), Aggregate Air Quality Index (AAQI) and Health Air Quality Index (HAQI) were used for comprehensive evaluation of severity of air pollution and its related health implications. The conventional AQI was developed and standardised by the United States Environmental Protection Agency to communicate the health significance of individual pollutants based on concentration thresholds and health categories [18]. The AQI classifications generally include the following categories: Good (0–50), Moderate (51–100), Unhealthy for Sensitive Groups (101–150), Unhealthy (151–200), Very Unhealthy (201–300), and Hazardous (>300), with increasing concern to the public health as the AQI increases.

However, the AQI has limitations in a multi-pollutant environment since it usually considers only the dominant pollutant at a specific time. To overcome this limitation Cairncross *et al*. (2007) [19] proposed the Aggregate Air Quality Index (AAQI) to combine the cumulative effects of various pollutants into a single air quality measure. Besides sub-indices of multiple pollutants, the AAQI also provides a more representative estimate of the total atmospheric pollution load in urban and industrial environment. The increment in the AAQI values means a higher risk of cumulative exposure and degradation of the ambient air conditions.

Air pollutants such as nitrogen dioxide (NO₂) are widely recognized as important indicators of urban air quality due to their adverse effects on human health and the environment [20]. Industrial activities are major contributors to ambient fine particulate matter (PM₂.₅) and heavy metal pollution, posing significant environmental and public health concerns in developing regions [21]. Long-term exposure to air pollution has been identified as a major risk factor for cardiovascular diseases, contributing substantially to the global burden of morbidity and mortality [22]. Satellite-based atmospheric observation systems have significantly improved air quality monitoring, enabling more effective forecasting and management of air pollution episodes [23]. Carbon monoxide (CO) concentrations exhibit seasonal variability influenced by meteorological conditions and emission sources, making continuous monitoring essential for effective air quality assessment [24]. Source apportionment studies have shown that both anthropogenic and natural sources contribute to fine and coarse particulate matter concentrations in urban environments [25]. The World Health Organization has established global air quality guidelines to minimize the health impacts associated with exposure to particulate matter, ozone, nitrogen dioxide, sulphur dioxide, and carbon monoxide [26]. Real-time air quality forecasting integrates atmospheric models, emission inventories, and meteorological data to support early warning systems and informed environmental decision-making [27].

Similarly, the Health Air Quality Index (HAQI) was developed to enhance the health-based interpretation of the exposure to air pollution, by explicitly incorporating the combined human health impacts of multiple pollutants rather than evaluating pollutants individually  [28]. The HAQI framework has been widely applied in epidemiological and environmental health studies to assess population-level exposure risk particularly in regions with chronic multi-pollutant pollution conditions. The HAQI, as opposed to the traditional AQI, can better reflect the synergistic effects of pollutants and the cumulative health risks of long-term exposure to the respiratory and cardiovascular systems.

We used the Aggregate and Health-Risk Adjusted (HAQI) metrics and other standards measures to check the quality of air. The results showed that particulate matter is the most important factor as its levels are greatly pronounced. Its dominance is high in the dry season and lowest in the wet season. Meteorological controls demonstrated the mechanisms of thermal stagnation and precipitation scavenging even when gaseous pollutants are at a normal level. HAQI shows a higher health risk. The results provide a seasonally adaptive, policy-relevant framework that conforms to WHO air quality guidelines.

## Materials and methods

Abeokuta (7.15^0^N, 3.35^0^E) is the capital city of Ogun State in the southwestern Nigeria with a population of about 608,000 persons, it is in the transition zone between tropical rainforest and savanna and is a quickly growing medium-sized city with a mix of residential, commercial, industrial and a huge traffic emission zone. There are two distinct seasons in its climate: the dry season from November to March and the wet season from April to October. Harmattan northeasterly winds transport Saharan mineral dust during the dry season, resulting in elevated particulate concentrations, reduced air quality, and impaired visibility. The wet season, on the other hand is mostly caused by moist south-westerly monson winds from the Atlantic Ocean. During the wet season, increased precipitation, enhanced atmospheric mixing, and wet deposition substantially influence ambient pollutant concentrations.

### Data collection

Hourly concentration of PM_2.5_, PM_10_, SO_2_, NO_2_, CO and O_3_ for January 2012 to December 2023 from the NASA Giovanni system using satellite observations (https://giovanni.gsfc.nasa.gov/giovanni/) and validated reanalysis products. The datasets provided consistent spatial and temporal coverage across the study period. Meteorological data for the same intervals were acquired from the Nigerian Meteorological Agency (NiMet) (https://nimet.gov.ng/).

These variables included air temperature, relative humidity, barometric pressure, wind speed, wind direction and humidity) (precipitation). All data sets were standardised to a uniform monthly resolution prior to analysis. Quality control consisted of data consistency checks, anomaly detection, and outlier screening before analysis. The meteorological data acquired from the Nigerian Meteorological Agency (NiMet) had no missing values hence no missing-data imputation was needed. These preprocessing procedures guaranteed the reliability of the pollutant–meteorological analyses performed in this study.

### Baseline data

Ground-level measurements were obtained using the ZN-MT10 9-in-1 Air Quality Detector and a locally fabricated air sampler. These compact, portable devices was deployed serving as a validation station for satellite retrievals.

### Statistical analysis

Seasonal patterns were examined alongside simple linear regression linking each pollutant with harvested meteorological variables. Each pollutant was tested separately against the six weather parameters to determine its mean response to specific atmospheric conditions at various months and periods of the year. These relationships were then subjected to regression slope test and coefficient of determination (R^2^) test to show coefficient of variance.

### Air quality indices

Three indices were used in this study to examine the pollutant levels and risk of exposure to human health. These indices are:

1. **The Air Quality Index (AQI):** this provides a single, dimensionless value that represents the overall air quality based on the concentration of individual pollutants as expressed in (1)

$$\begin{array}{c} I_{p}=\frac{I_{HI}-I_{LO}}{BP_{HI}-BP_{LO}}\times \left(C_{p}-BP_{LO}\right)+I_{LO} \end{array}$$ (1)

where:

- $I_{p}$= AQI sub-index for pollutant *p*
- $C_{p}$= observed pollutant concentration
- $BP_{HI}$ and $BP_{LO}$= upper and lower breakpoint concentrations surrounding $C_{p}$
- $I_{HI}$ and $I_{LO}$= corresponding AQI values for the upper and lower breakpoints

2. **Aggregate Air Quality Index (AAQI):** the AAQI was calculated using the rot-sum- square method to represent the combined pollution levels of different pollutants and in this case six (6) pollutants. (2) shows the presentation

$$\begin{array}{c} AAQI=\left(\sum_{i}^{n}{\left(AQI\right)}_{I}^{P}\right)\frac{\text{1}}{P} \end{array}$$ (2)

Where P is the aggregate coefficient or weighting exponent used to control the sensitivity of the AAQI to higher pollutant concentrations.

3. **Health-Risk Adjusted AQI (HAQI):** The Health-risk adjusted AQI was then created to include pollutants-specific risk factors, which combined each pollutants impact on overall health. The HAQI was calculated using the method described by [5] which integrates WHO risk coefficient for both short- and long-term exposures.

$$\begin{array}{c} HAQI=\left(\sum_{I}^{N}\frac{C_{I}}{\text{1}\text{0}}\right) \end{array}$$ (3)

Where:

- ${C\;}_{I}$ = Concentration air pollutant the term $C_{i}\;$ represents the concentration of pollutant i.

These indices were computed on monthly basis, thereby enabling the seasonal assessment of both pollution concentration and associated health risk. The analytical process was executed using Python and R statical software. To effectively show the responsiveness of the pollutants to meteorological influences, and the explicitly show the seasonal trends, the data set visualisation methods such as scatterplots, seasonal time series analysis and regression diagnostics were implemented.

### Pollutant projections

The Seasonal Autoregressive Integrated Moving Average (SARIMA) model was used to study the random, trend, and seasonal changes that happen in monthly air pollution time series. SARIMA adds seasonal autoregressive and moving average structures to the ARIMA framework. It is written as $SARIMA(p,d,q)(P,D,Q{)}_{s}$, where p, d, and q are non-seasonal orders, P, D, and Q are seasonal orders, and s is the seasonal periodicity (12 for monthly data). The general form of multiplication looks like this:


$$\begin{array}{c} \Phi_{P}(B^{s})\phi_{p}(B)(\text{1}-B{)}^{d}(\text{1}-B^{s}{)}^{D}Y_{t}=\Theta_{Q}\left(B^{s}\right)\theta_{q}(B)\varepsilon_{t} \end{array}$$ (4)

where B is the backshift operator ($BY_{t}=Y_{t-\text{1}}$) and $\varepsilon_{t}\sim WN(\text{0},\sigma^{\text{2}})$. Non-seasonal differencing $(\text{1}-B{)}^{d}\;$removes deterministic trends, while seasonal differencing $(\text{1}-B^{s}{)}^{D}\;$eliminates annual periodicity. The autoregressive polynomials $\phi_{p}(B)$and $\Phi_{P}\left(B^{s}\right)\;$model temporal persistence and inter-annual dependence, whereas the moving average polynomials $\theta_{q}(B)\;$and $\Theta_{Q}\left(B^{s}\right)\;$show how random shocks spread. We used the Augmented Dickey–Fuller test and visual checks to see if the data was stationary. When necessary, differencing was used.

We used the Box–Jenkins method to find the model by looking at the ACF/PACF and lowering the Akaike (AIC) and Bayesian (BIC) information criteria. We used Maximum Likelihood Estimation to find the parameters, assuming that the innovation was Gaussian. Residual diagnostics, including the Ljung–Box Q-statistic, were employed to verify the adequacy of the model. We used conditional expectations ${\hat{Y}}_{t+h|t}=E(Y_{t+h}|F_{t})$,to make predictions repeatedly. We also used asymptotic variance estimates to make prediction intervals. We picked the SARIMA framework because it can show linear stochastic dependence, seasonal atmospheric repetition, and short-term pollutant persistence in a way that is statistically sound for making predictions. Hyndman and Athanasopoulos [29] describe SARIMA as an extension of the ARIMA framework that incorporates seasonal autoregressive and moving-average components.

## Results and discussion

This section examines the variations in air pollution levels in Abeokuta between 2012 and 2023, highlighting periods of elevated concentrations linked to causes and seasonal changes. Temporal patterns were identified from the distribution of pollutant levels across the study years. Relationships between each pollutant and temperature, rainfall, windspeed, pressure, wind direction and humidity were also assessed. These interactions explain how weather conditions promote dispersion, buildup, or removal of pollutant concentrations in Abeokuta.

### PM_2.5_ and Meteorological parameters

**Fig 1:**
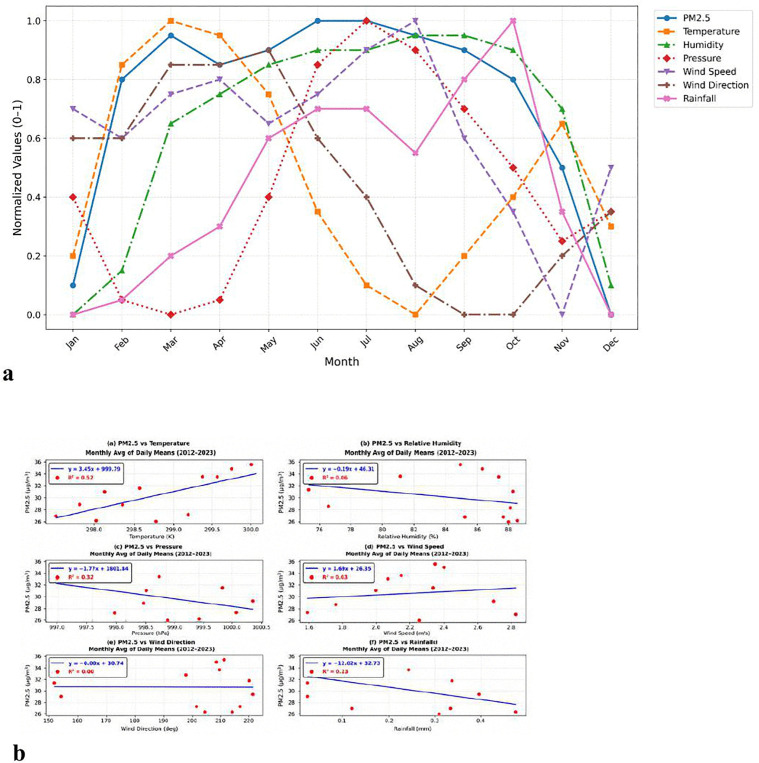
a: Monthly average plots of PM_2.5_ in relation to various meteorological parameters. **b**: Monthly average correlation of PM_2.5_ with various meteorological parameters.

Fig 1a shows that during the dry season (December to February), when the Harmattan dust is moving and the atmosphere is stable, PM_2_._5_ concentrations reached their annual maxima during the dry season. However, increased rainfall and convective activity during the wet season promote wet scavenging and atmospheric dispersion, resulting in lower PM_2_._5_ concentrations. [30,31,32].

Fig 1b showed that for PM_2.5_ there is a moderate positive correlation with temperature (R² = 0.52) and a strong negative correlation with rainfall (R² = 0.23). Relative humidity has a weak negative correlation (R² = 0.06) which means that the reduction of PM_2.5_ happens after humidity levels are high enough for precipitation scavenging to happen. Elevated atmospheric pressure is associated with reduced vertical mixing and enhanced pollutant accumulation, which leads to more accumulation of PM_2.5._ on the other hand, low pressure systems during the monsoon season speed up dilution. Wind-direction patterns indicate the influence of continental dust transport from the northeast and cleaner maritime air masses from the southwest.

### PM_10_ and Meteorological Parameters

**Fig 2:**
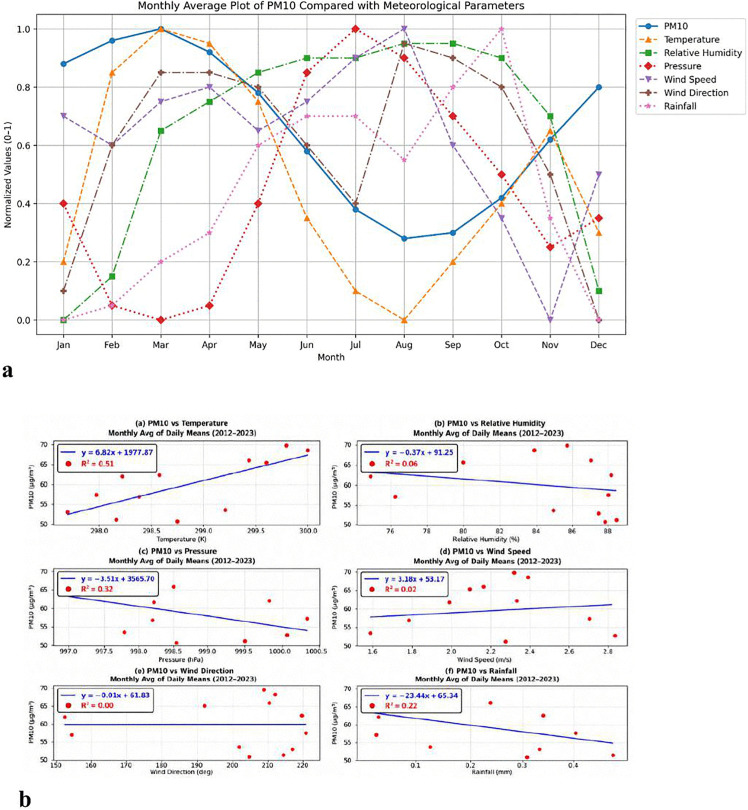
a: Monthly average plots of PM_10_ compared to various meteorological parameters. **b**: Monthly average correlation of PM_10_ with various meteorological parameters.

PM_10_ exhibits a stronger seasonal cycle compared to PM_2.5_ as seen in Fig 2a. It slowly rises from December to March then drops sharply during the months with most rainfall. The elevated PM₁₀ concentrations observed during the dry season are primarily attributable to dust resuspension and long-range Saharan dust transport. Temperature has a small positive effect (R² = 0.51), rainfall exhibited the strongest association with PM₁₀ removal through wet deposition processes, relative humidity and wind speed exhibit week correlation (R² ≈ 0.06 and 0.02), these findings suggest that seasonal air-mass transport exerts a greater influence on PM₁₀ variability than local-scale turbulent mixing.

### NO_2_ and Meteorological parameters

**Fig 3:**
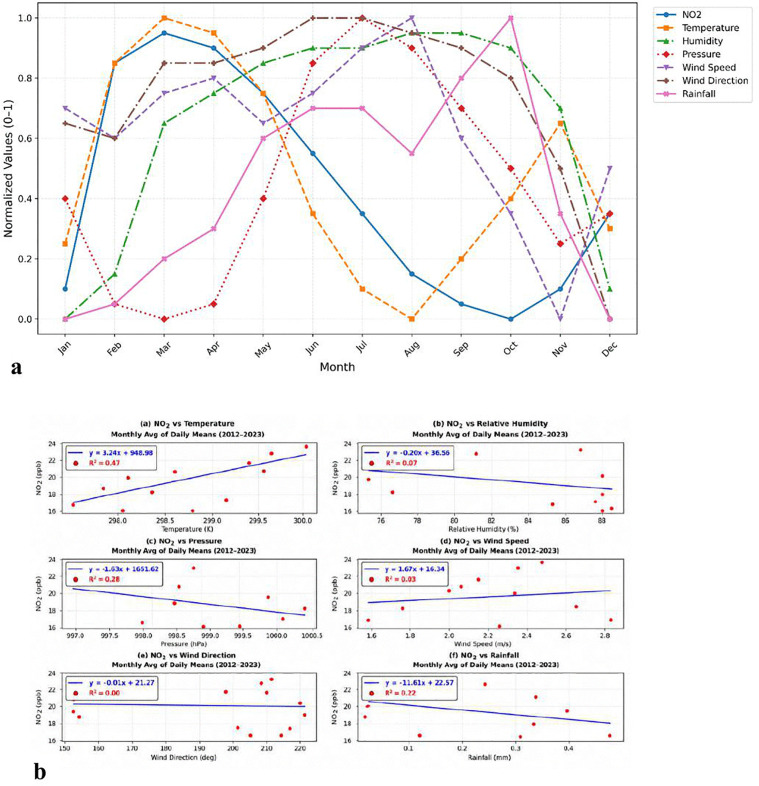
a: Monthly average graphs of NO_2_ plotted against meteorological parameters. **b**: Monthly average correlation of NO_2_ with various meteorological parameters.

The levels of NO_2_ are highest in the dry months and lowest in the rainy months as seen in Fig 3a. This reduction may be attributed to enhanced wet deposition and increased photochemical transformation during the wet season. There is moderate positive correlation (R² = 0.47) between temperature and emissions as seen in Fig 3b. This means that the positive association with temperature suggests enhanced photochemical activity and secondary pollutant formation under warmer conditions. Relative humidity (R² = 0.07) and rainfall (R² = 0.22) have opposite effect, just like scavenging processes do.

Pressure has a negative control (R² = 0.28), which means that stable subsidence conditions during the dry season favour pollutant accumulation by suppressing atmospheric dispersion.

Wind factors have minimal influence, therefore the variations in NO_2_ concentrations appear to be primarily driven by local combustion-related emission sources. The average NO_2_ levels (about 21.3 ppb) are still below the WHO’s hourly limits of (106 ppb), but there is a risk of exposure during dry seasons.

### CO and Meteorological Parameter

**Fig 4:**
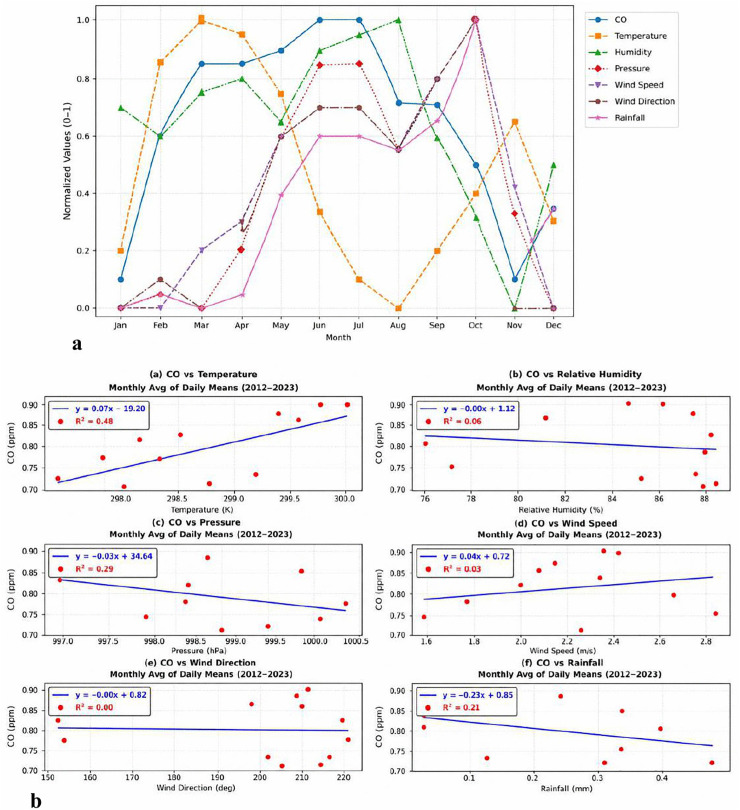
a: Monthly average plots of CO against different Meteorological Parameters. **b**: Monthly average correlation of CO with various meteorological parameters.

Elevated CO concentrations during the late dry season are likely associated with increased combustion activities and reduced atmospheric ventilation. (March to April). Vertical mixing and the removal of precipitation cause concentrations to drop quickly during the wet season as seen in Fig 4a. Fig 4b showed temperature has a moderate positive correlation (R² = 0.48). The effect of rain on removal is shown by R² = 0.21. Relative humidity has a minor impact on concentrations (R² = 0.06).

The effect of wind speed is small (R² = 0.03), which means that emissions from the area are the most important. Northeasterly continental air masses may facilitate regional transport of CO during the dry season, while maritime monsoon conditions are associated with enhanced atmospheric mixing and reduced CO accumulation.

### O_3_ and meteorological parameters

**Fig 5:**
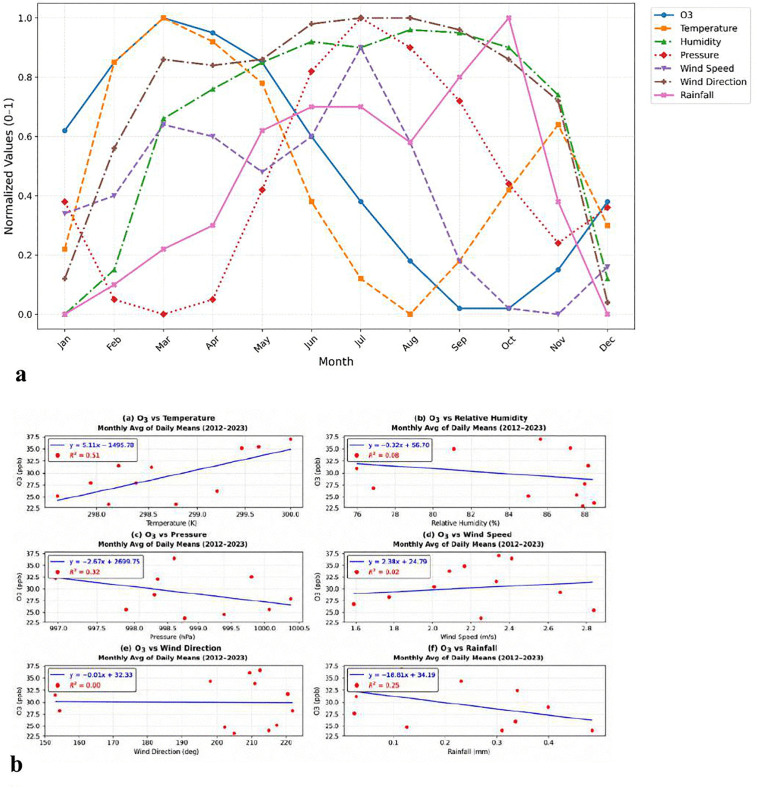
a: Monthly average plots of O_3_ versus various meteorological parameters. **b**: Monthly average correlation of O_3_ with various meteorological parameters.

During the late dry season (February to April), O_3_ concentrations reached their highest values during periods of enhanced solar radiation. During the cloudy monsoon months, they drop as seen in Fig 5a. The temperature exhibited a moderate positive association with O_3_ formation. (R² = 0.51), which backs up the idea that NOx and VOCs can make O_3_ through photochemical processes. Relative humidity has a weak effect on formation (R² = 0.08). Increased cloud cover and precipitation reduce O_3_ concentrations by limiting photochemical production and enhancing atmospheric removal processes.

Pressure has the opposite effect on O_3_ (R² = 0.32), which shows that regional circulation is happening according to Fig 5b. There are seasonal effects that are easy to see, but wind speed and direction do not have much of an effect on statistics.

### SO_2_ and Meteorological Parameter

**Fig 6:**
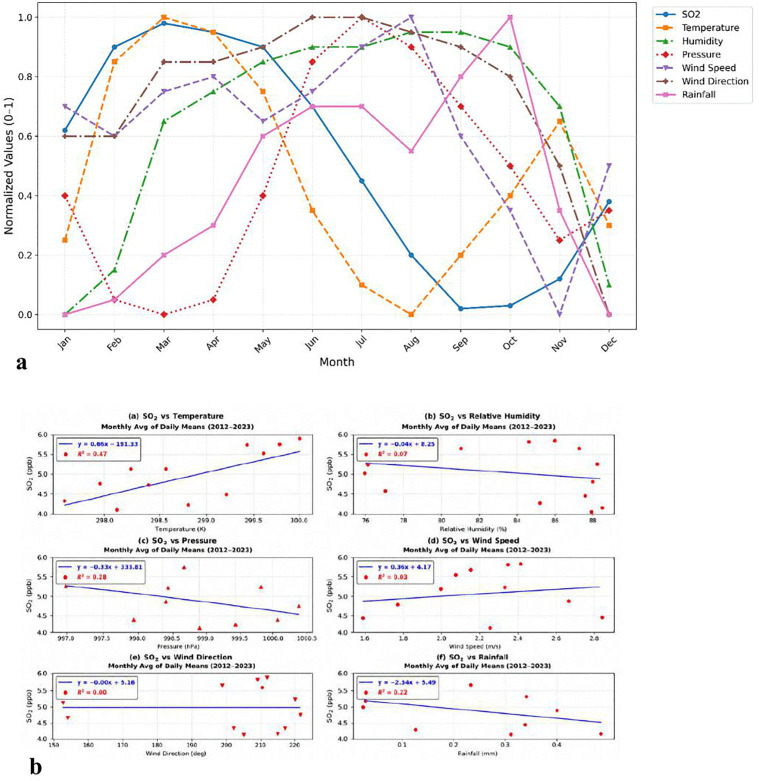
a: Monthly average plots of SO_2_ versus various meteorological parameters. **b**: Monthly average correlation of SO_2_ with various meteorological parameters.

**Fig 7:**
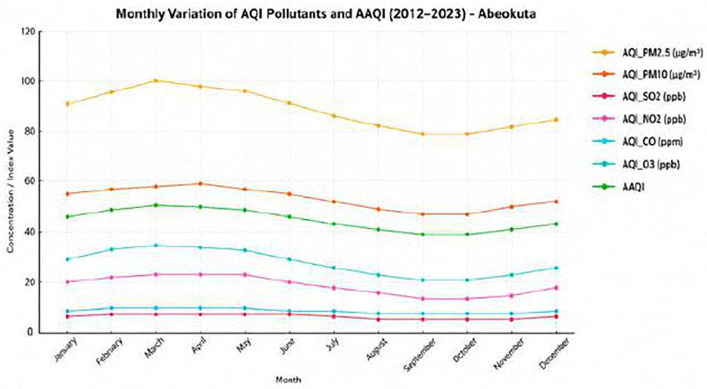
Monthly changes in AQI in each pollutant and an overall AAQI.

The Fig 6a shows elevated SO_2_ during the dry months. The observed increase in SO_2_ concentrations may be associated with emissions from generator use, vehicular activities, and biomass combustion. The temperature has a small effect on concentrations (R² = 0.47), relative humidity has a small effect on SO_2_ (R² = 0.07), but rainfall has a big effect on pollutants through wet deposition (R² ≈ 0.22). Pressure and dispersion are related in the opposite way (R² = 0.28) this is visible on Fig 6b. Wind-related variables exhibited relatively weak correlations with SO_2_ concentrations, which means that emissions in the area come from more than one source instead of just one Fig 7.

### The Monthly Air Quality Index and Aggregate Air Quality Index for Abeokuta from 2012 to 2023

The monthly analysis of the Air Quality Index (AQI) shows that PM_2_._5_ was the dominant contributor to overall air-quality degradation in Abeokuta. Harmattan dust transport, biomass burning, and weak atmospheric dispersion make it peak between February and April. PM₁₀ has a similar peak during the dry season, which happens when coarse particles and soil dust are stirred up again. But it drops a lot during the wet season because of wet deposition. SO_2_ is usually low, but it goes up a little during the dry months, probably because of burning fuel. NO_2_ levels also rise during the late dry season because vehicular emissions are a significant source of NO_2_ during the late dry season and the boundary layer is not mixing as much. Elevated NO_2_ exposure has been associated with increased cardiovascular and respiratory health risks. [33, 34,1,35].

CO concentrations were generally lower during the wet season, likely due to enhanced atmospheric dispersion and dilution. When it is dry, the levels are highest because the combustion isn’t complete. On the other hand, O_3_ levels go up in the late dry season because the strong sunlight speeds up the process of photochemical formation. These pollutants contribute to increases in the Aggregate Air Quality Index (AAQI) from December to April, with the highest levels in March and April. During the wet season, rain lowers concentrations by mixing with the air and taking up space. The fact that both particulate and gaseous pollutants are rising at the same time means that the concurrent elevation of multiple pollutants indicates increased multi-pollutant exposure. The dry season is when public health is most at risk, which shows how important it is to have health warnings and targeted emission controls.

**Fig 8:**
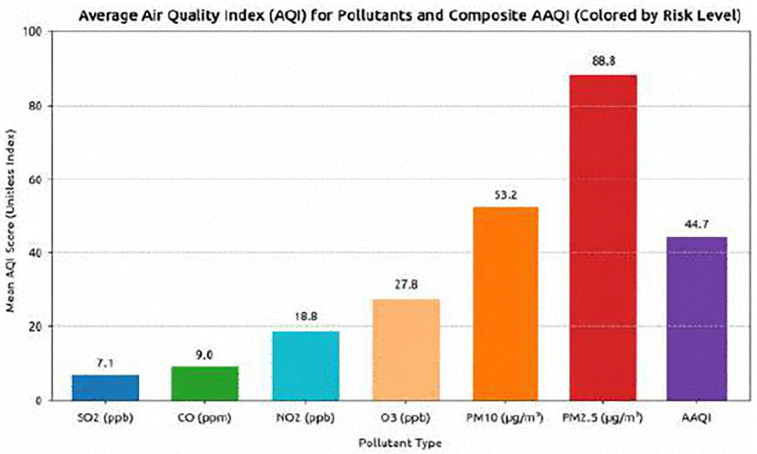
Average AQI Scores for each pollutant from 2012 to 2023.

Fig 8 above shows that PM_2_._5_ has the highest AQI value (about 88.8), which means it is the main cause of air-quality risk in Abeokuta. PM₁₀ (about 53.2) also gets to unhealthy levels, which shows how big of an effect particulate pollution has. The composite AAQI (about 44.7) shows that there is a moderate amount of pollution overall, mostly from particles and not gases. Gaseous pollutants like SO_2_, CO, NO_2_, and O_3_ are still low, which means they do not significantly elevate the health risk as much as particulates do. Fine and coarse particles are the main things that affect public health risk in Abeokuta.

### The AAQI pattern and its effects on public health

The majority of AAQI values fell within the WHO moderate risk category (46–51), suggesting that sensitive individuals may experience respiratory discomfort and other adverse health effects during periods of high pollution. The coincidence of the maximum values of AAQI with maximum values of PM_2.5_ and PM_10_ emphasises the dominant contribution of PM to the overall air-quality deterioration in Abeokuta. This pattern indicates that particulate pollution is the main source of air quality related health risk in the study area and it is mainly from local and regional emission sources such as vehicle exhaust, biomass burning, resuspended road dust from unpaved roads and Harmattan Saharan dust transport [36,8]. Generally, gaseous pollutants had lower AQI values, but their possible health implications should not be neglected. SO_2_ and CO contributed little to the total air-quality burden, while NO_2_ and O_3_ had non-negligible impacts related to traffic-related emissions and photochemical processes, respectively [11].

In addition, the meteorological environment plays an important role in the accumulation, dispersion and transformation of pollutants, further affecting the levels of air-quality and its associated health risks. The seasonal variations of the air quality over Abeokuta are due to the temperature, rainfall, atmospheric stability, humidity and wind characteristics that influence the transport and removal processes of pollutants.

Table 1 shows Health-Risk Classification of the Health-Based Air Quality Index (HAQI) Using WHO Guideline Reference Levels while Table 2 shows HAQI values range from about 68 during the rainy season to about 98 during the late dry season for Abeokuta. This means that the time of year when health risks are highest is between January and April. Previous studies have consistently reported strong associations between particulate matter exposure and adverse cardiovascular and respiratory health outcomes. [37,38,39,4,40,41,42];. PM_2_._5_ has the strongest effect on health (HR ≈ 1.7–2.3), and PM₁₀ has the second strongest effect (HR ≈ 1.1–1.6) [6,5,17]. During the dry season, gaseous pollutants like NO_2_, CO, O_3_, and SO_2_ also reach their highest levels. This can cause inflammation in the airways and stress on the heart.

**Table 1:** Health-Risk Classification of the Health-Based Air Quality Index (HAQI) Using WHO Guideline Reference Levels.

HAQI Value	Health Category	WHO Standard Meaning	Interpretation in Study
0 – 25	Clean/ Ideal	All pollutants within WHO guideline levels	No measurable health risk
26 – 50	Acceptable	Near WHO limits	Very low risk
51 – 100	Slight Pollution	At least one pollutant exceeds WHO guideline	Sensitive individuals affected
101 – 150	Moderate Pollution	Multiple pollutants exceed WHO limits	Noticeable respiratory effects
151 – 200	Unhealthy	Significant combined exposure	Increased hospital admissions
201 – 300	Very Unhealthy	Strong multi-pollutant interaction	Serious health impact
>300	Hazardous	Extreme exposure	Public health emergency

Source: Adapted from World Health Organization [1] and United States Environmental Protection Agency (2023).

**Table 2:** Monthly HAQI for Abeokuta (2012 - 2023).

Month	HAQI	HR_PM_2.5_ (µg/m)	HR_PM_10_ (µg/m)	HR_SO_2_ (ppb)	HR_NO_2_ (ppb)	HR_CO (ppm)	HR_O_3_ (ppb)
January	86.74	2.09	1.39	0.13	0.85	0.21	0.53
February	93.49	2.23	1.49	0.14	0.94	0.22	0.59
March	97.42	2.32	1.55	0.15	0.99	0.22	0.62
April	97.33	2.31	1.55	0.15	0.99	0.22	0.62
May	93.53	2.23	1.49	0.14	0.94	0.22	0.59
June	86.81	2.09	1.39	0.13	0.85	0.21	0.53
July	78.86	1.92	1.28	0.12	0.75	0.19	0.47
August	71.88	1.77	1.18	0.11	0.66	0.18	0.41
September	67.83	1.68	1.12	0.1	0.61	0.18	0.38
October	67.91	1.69	1.12	0.1	0.61	0.18	0.38
November	71.83	1.77	1.18	0.11	0.66	0.18	0.41
December	78.61	1.91	1.28	0.12	0.75	0.19	0.47

When several pollutants go up at the same time, it means that they are toxic in a way that makes them work together and build up, not just on their own. From July to October, rain and mixing in the air lower hazard ratios, which gives some seasonal protection [43,44,45,46]. The air quality in Abeokuta changes a lot with the seasons. This is mostly because the temperature stays the same, there isn’t much rain, and the atmosphere is stable. During the dry season, the main sources of exposure are traffic, burning biomass, and dust that gets stirred up again. The pollution in the background shows that there is still a health risk even when it’s not the busiest time of year. The results indicate the need for policy interventions that change with the seasons to cut down on pollution and health warnings for groups that are at risk. Future efforts to improve air quality while taking climate into account should include exposure modelling, source apportionment, and advanced prediction frameworks [30,47,48].

### Long-Term Projection of PM_2.5,_ PM_10_, NO_2_, CO, O_3_, SO_2_ Concentrations (2024–2034) for Abeokuta

**Fig 9:**
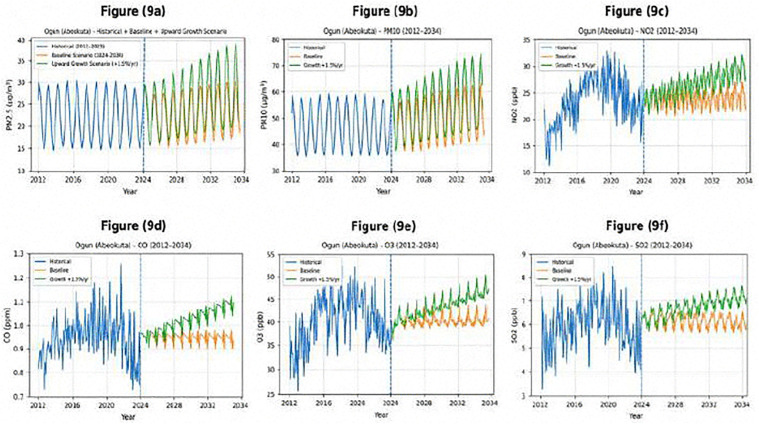
Historical, Baseline, and Growth Scenario Projections for Abeokuta, 2012–2034.

Fig 9a above shows the upward growth scenario (+1.5% per annum) demonstrates potential escalation in pollutant levels under increased emission intensity and urban expansion. Comparative analysis of PM_2.5_ in Abeokuta indicates that sustained growth could significantly elevate long-term exposure risks, with implications for public health, environmental quality, and regulatory policy. The divergence between baseline and growth projections underscores the necessity for emission mitigation strategies and sustainable urban planning. Fig 9b showed the upward growth scenario (+1.5% per annum) of PM_10_ demonstrates the potential escalation in particulate concentration under continued urban expansion and anthropogenic pressure.

The divergence between the baseline and growth curves highlights the increasing long-term exposure risk and underscores the importance of emission control policies, air quality monitoring, and sustainable urban planning strategies.

The baseline projection (2024–2034), obtained using SARIMA modelling as seen in Fig 9c assumes persistence of historical seasonal characteristics without structural emission shifts. The growth scenario (+1.5% per annum) represents a potential intensification in nitrogen oxide emissions under continued urban expansion and energy demand. The widening gap between the baseline and growth curves highlights increased future exposure risks, particularly in high-density urban environments. The divergence between the baseline and growth curves of CO in Abeokuta in Fig 9d indicates that without mitigation strategies, long-term exposure may increase progressively. These projections emphasize the importance of cleaner combustion technologies, improved transport policies, and stricter emission regulation frameworks.

The baseline projection (2024–2034) of O_3_, generated using SARIMA modelling (Fig 9e) maintains historical seasonal dynamics while assuming no structural emission shifts. The + 1.5% annual growth scenario reflects potential enhancement in precursor emissions and urban expansion, resulting in elevated tropospheric ozone formation. The increasing separation between baseline and growth trajectories suggests heightened long-term exposure risk, with implications for respiratory health, ecosystem stability, and air quality management strategies. SARIMA modelling of Fig 9f maintains historical seasonal behaviour while assuming no structural emission change. The + 1.5% annual growth scenario represents a potential intensification in Sulphur emissions driven by industrial expansion and energy demand. The increasing divergence between baseline and growth trajectories indicates rising long-term exposure risks in the absence of mitigation policies. These projections underscore the importance of Sulphur content regulation in fuels, adoption of cleaner industrial technologies, and strengthened environmental compliance frameworks to protect public health and atmospheric quality.

### Data validation

**Fig 10:**
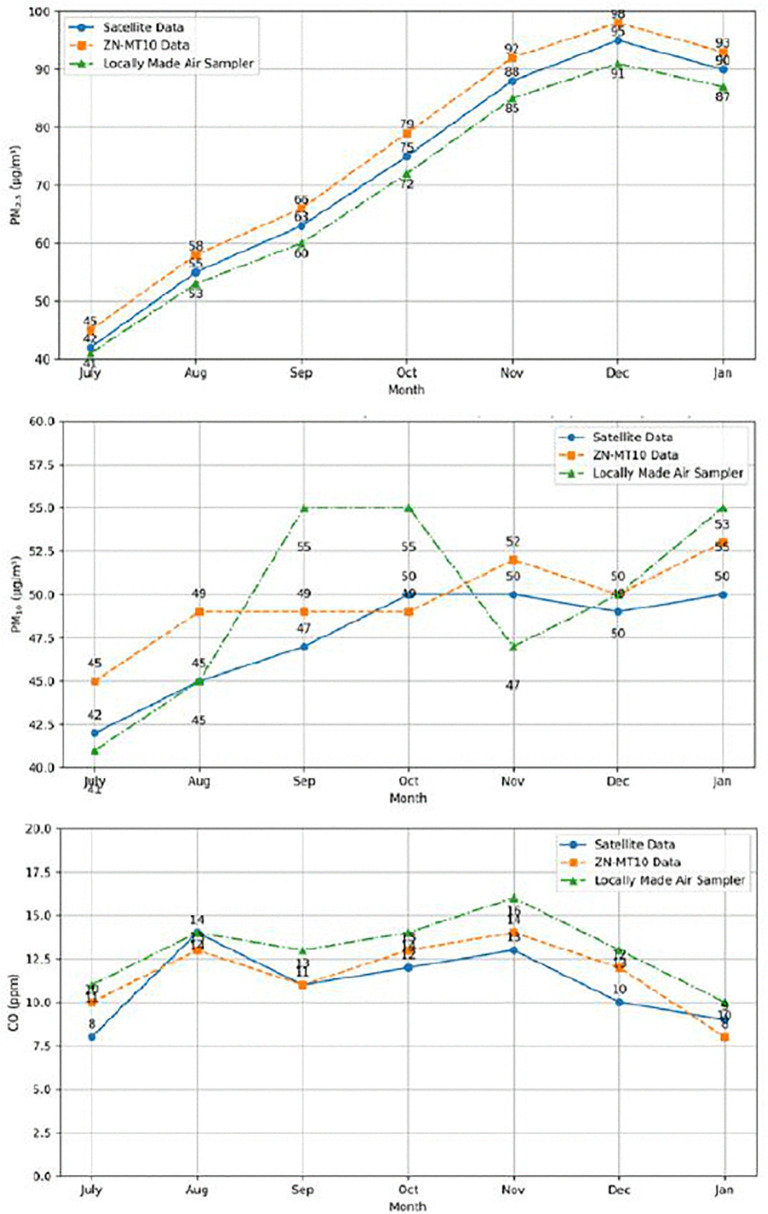
Comparative Analysis of Satellite, ZN-MT10, and Locally Made Air Sampler Measurements for PM_2_._5_, PM_10_, and CO.

To validate the satellite data used for this study, 2 ground-based air samplers (ZN-MT10 air sampler) and a locally fabricated air sampler were used to make observations for 7 Months (July 2025 to January 2026) as seen in Fig 10.

Fig 10 showed the level of correlation between the Satellite data, ZN-MT10, and Locally Made Air Sampler for PM_2_._5_, PM₁₀, and CO as all instruments could measure these pollutants as side other differentials. The results from Fig 10 largely validated obtained satellite data.

### The diurnal variation of pollution concentrations using satellite Data, ZN-MT10 and Locally Made Air samplers

The diurnal variation of PM concentrations across the months exhibits a consistent bimodal pattern, with pronounced peaks occurring during early morning (06:00–08:00) and evening hours (18:00–21:00). These peaks are attributed to reduced atmospheric mixing, temperature inversion conditions, and increased anthropogenic activities such as traffic and domestic combustion. Conversely, minimum concentrations are observed during midday (12:00–15:00), corresponding to enhanced solar radiation and boundary layer development, which promote pollutant dispersion. The locally developed air sampler consistently reports slightly lower concentrations compared to satellite and ZN-MT10 measurements, though all instruments exhibit similar temporal trends, indicating strong agreement in capturing diurnal variability.

**Fig 11:**
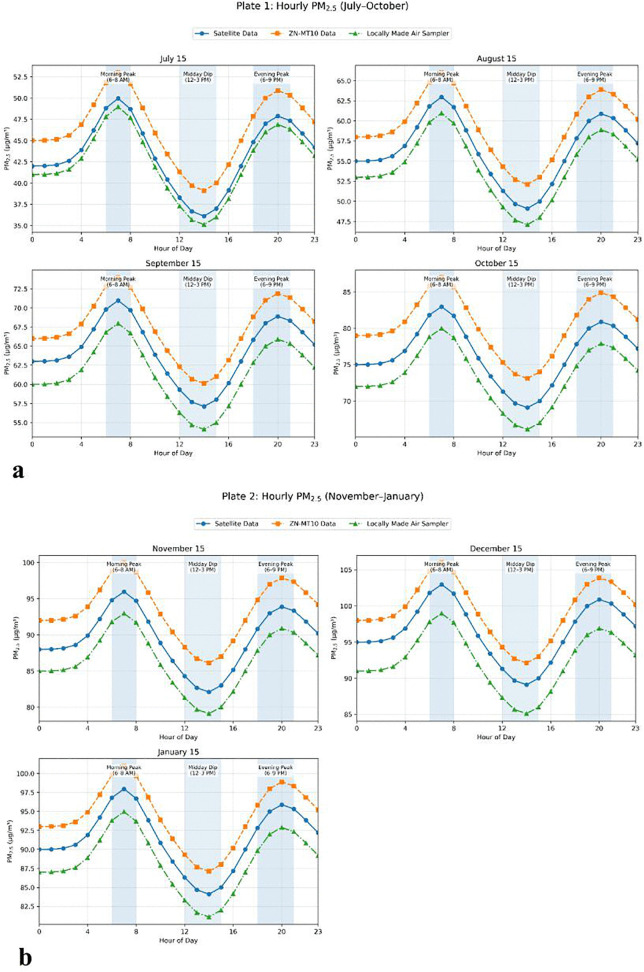
a: The diurnal variation of PM_2_._5_ concentrations. b: The diurnal variation of PM_2_._5_ concentrations.

The hourly profiles for the 15th day of each month as seen in Fig 11 (a and b) shows a consistent diurnal structure across the three measuring instruments. With elevated PM_2.5_ concentrations during the early morning hours and evening periods and lower levels around midday. This pattern is consistent with nocturnal and early-morning atmospheric stability, shallow boundary-layer height, traffic and domestic combustion emissions, followed by enhanced midday vertical mixing and dispersion under stronger solar heating. Across all months on the 15^th^ day, the ZN-MT10 generally records the highest concentrations, the locally made air sampler the lowest, while the satellite-derived values remain intermediate. The strong agreement in hourly curves suggests that all three instruments capture the same temporal behaviour despite differences in magnitude.

**Fig 12:**
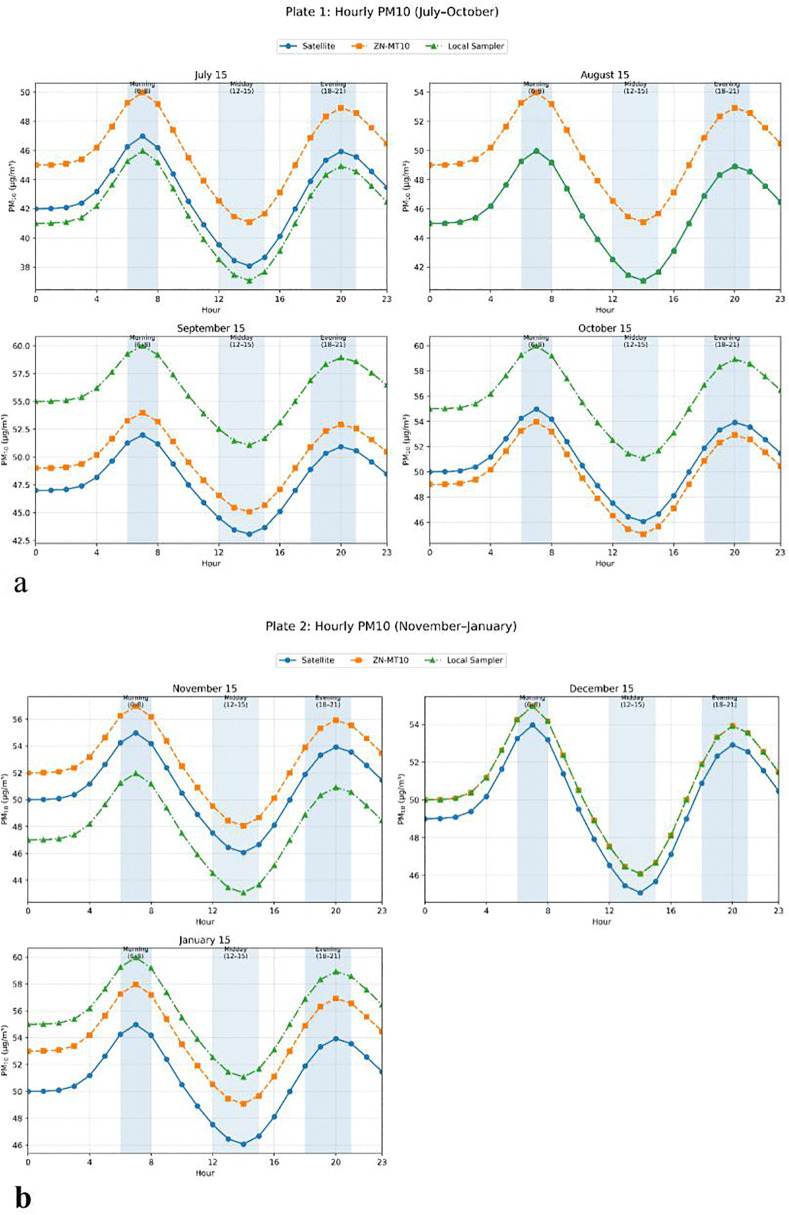
a: The diurnal variation of PM_10_ concentrations. b: The diurnal variation of PM_10_ concentrations.

PM₁₀ has a daily cycle that is similar to PM_2_._5_, according to Fig 12 (a and b) but the amplitude is a little lower. This is because PM₁₀ is more closely related to mechanical processes (like dust resuspension and wind) than to combustion sources. The midday dip is not as strong as it is for PM_2_._5_, which means that coarse particles are less affected by boundary layer dilution.

**Fig 13:**
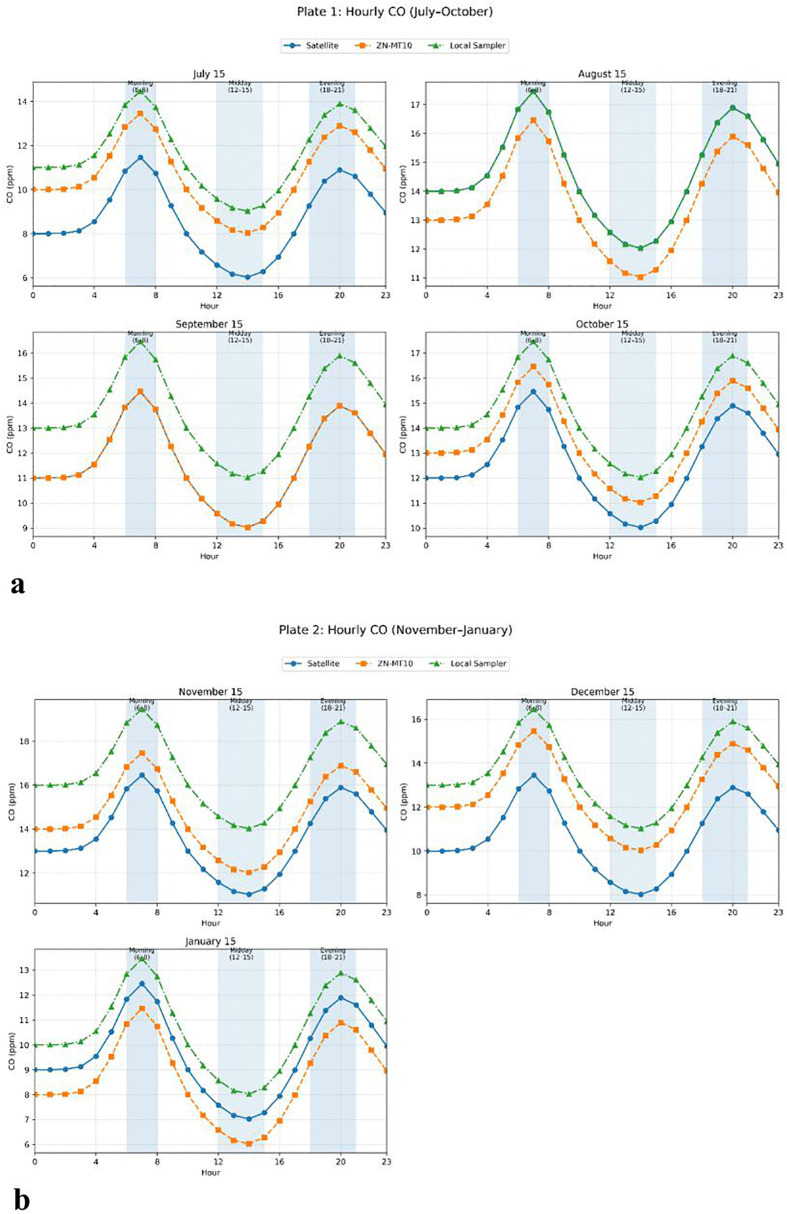
a: The diurnal variation of CO concentrations. b: The diurnal variation of CO concentrations.

The levels of CO change every day in a clear bimodal pattern as shown in Fig 13 (a and b) with the highest levels in the morning and evening. This is because of emissions from cars, heating homes, and steady weather. The drop in CO at noon is not as noticeable as the drop in particles. This is because CO stays in the atmosphere longer and does not depend as much on boundary layer dilution alone. The fact that there are high levels of CO in the air at night shows trapping of CO from the sources of emissions, like generators and vehicular activities and little transport or diffusion.

### Limitations and future research

Some limitations need to be considered while the long-term pollutant–meteorological interactions in Abeokuta were well evaluated. First, the analysis was mostly based on long-term monthly averages from the 2012–2023 observations. This approach can be effective in identifying persistent seasonal and climatological patterns but may smooth over short-term pollution episodes, extreme events and potential interannual variations associated with changes in emissions, meteorological anomalies, or urban development. Therefore, the results should be regarded as indicative of average long-term conditions, not of pollution behaviour in a certain year. Second, the study used satellite-derived and area averaged datasets that may not capture fine scale spatial heterogeneity within Abeokuta. This may lead to under-representing localised pollution hotspots related to the main traffic corridors, industrial activities, residential combustion, open waste burning and land use differences. Third, the meteorological controls were examined using the regression-based relationships, but other factors such as changes in emission inventories, socio-economic growth and urban growth were not explicitly incorporated into the analysis. In future studies, the combination of high-resolution ground-monitoring networks, source apportionment techniques and spatially resolved modelling approaches should be used to investigate in more detail intra-urban variability, extreme pollution episodes and long-term temporal trends.

## Conclusion

The results indicate that the air quality in Abeokuta is largely determined by seasonal meteorological conditions. The dry season favours the accumulation of pollutants through atmospheric stability; transport of dust and enhanced emissions related to combustion. The wet season favours the removal of pollutants through rainfall and atmospheric mixing. Particulate matter was found to be the most significant contributor to degrading air quality and health risks, and thus the most important air pollution problem in the study area. These results highlight the combined impact of regional atmospheric processes and local emission sources and underscore the importance of seasonally targeted air-quality management strategies.

Although the focus here is on Abeokuta, the pollutant-meteorology interactions documented are representative of many rapidly growing tropical cities in West Africa and beyond in the developing world and emphasise the generalisability of seasonally based air-quality management strategies in environments perturbed by biomass burning, urban emissions, and strong wet-dry climatic cycles.

The findings of this study offer a practical framework for seasonally adaptive air-quality management in Abeokuta and similar fast urbanising tropical cities. Overall, the AAQI was dominated by PM_2.5_ and PM_10_, suggesting that particulate matter should be the focus of pollution-control interventions. Regulatory agencies should emphasise measures to reduce emissions from traffic, open waste burning, biomass combustion and resuspended road dust during dry season when particulate levels and associated health risks are at their peak. These measures may include stricter enforcement of vehicle emission standards, restrictions on open burning, improved road maintenance and dust suppression programmes and promotion of cleaner household and commercial energy sources.

The strong impact of meteorological conditions on pollutant accumulation also indicates the importance of meteorology-based air quality forecasting and early warning systems. Public-health advisories could be issued on the basis of seasonal forecasts and real-time monitoring, especially for vulnerable populations, such as children, older adults and those with respiratory or cardiovascular conditions, during periods of elevated pollution. Furthermore, the observed pollutant–meteorology relationships provide a scientific basis for incorporating air-quality considerations into urban planning, transportation management, and environmental-health policies. Collectively, these measures contribute to a proactive, seasonally responsive approach to pollution management that can reduce population exposure, improve public health outcomes, and promote sustainable urban development.

## Supporting information

S1 Appendix AQI Categories. According to the WHO, the AAQI and AQI range for air quality for each pollutant in this study are summarized from Tables A to F below. Table A: Air Quality Index (AAQI) Classification for PM2.5 (µg/m³) Concentrations. Table B: Air Quality Index (AAQI) Classification for PM10 (µg/m³) Concentrations. Table C: Air Quality Index (AAQI) Classification for NO2 (ppb) Concentrations. Table D: Air Quality Index (AAQI) Classification for SO2 (ppb) Concentrations. Table E: Air Quality Index (AAQI) Classification for CO (ppm) Concentrations. Table F: Air Quality Index (AAQI) Classification for O3 (ppb) Concentrations.
(XLSX)
